# Pseudoalteromone A, a Ubiquinone Derivative from Marine *Pseudoalteromonas* spp., Suppresses Melanogenesis

**DOI:** 10.3390/md19110612

**Published:** 2021-10-28

**Authors:** Su-Jin Lim, Dae-jin Min, Sohee Kim, Jihye Lee, Eun-Soo Lee, Hyuk Kim, Sung-Yoen Cho, Heung-Soo Beak, Chang-Seok Lee, Sang-Jip Nam, Jaeyoung Ko

**Affiliations:** 1 Department of Beauty and Cosmetic Science, Eulji University, Seongnam 13135, Korea; jimnote91@naver.com; 2 Basic Research & Innovation Division, Amorepacific R&D Unit, Yongin 17074, Korea; djmin@amorepacific.com (D.-j.M.); soopian82@gmail.com (E.-S.L.); atharabia@amorepacific.com (H.K.); csy123@amorepacific.com (S.-Y.C.); monmimi@amorepacific.com (H.-S.B.); 3 The Graduate School of Industrial Pharmaceutical Sciences, College of Pharmacy, Ewha Womans University, Seoul 03760, Korea; chi0736@naver.com; 4 Department of Chemistry and Nanoscience, Ewha Womans University, Seoul 03760, Korea; jl3414@gmail.com; 5 Laboratories of Marine New Drugs, REDONE Seoul, Seoul 08594, Korea

**Keywords:** *Pseudoalteromonas* sp., pseudoalteromone A, marine natural product, melanogenesis

## Abstract

An ubiquinone derivative, pseudoalteromone A (**1**), has been isolated from two marine-derived *Pseudoalteromonas* spp., APmarine002 and ROA-050, and its anti-melanogenesis activity was investigated. The anti-melanogenic capacity of pseudoalteromone A was demonstrated by assessing the intracellular and extracellular melanin content and cellular tyrosinase activity in the B16 cell line, Melan-a mouse melanocyte cell line, and MNT-1 human malignant melanoma cell line. Treatment with pseudoalteromone A (40 μg/mL) for 72 h reduced α-melanocyte-stimulating hormone (α-MSH)-induced intracellular melanin production by up to 44.68% in B16 cells and 38.24% in MNT-1 cells. Notably, pseudoalteromone A induced a concentration-dependent reduction in cellular tyrosinase activity in B16 cell, and Western blot analyses showed that this inhibitory activity was associated with a significant decrease in protein levels of tyrosinase and tyrosinase-related protein 1 (Tyrp-1), suggesting that pseudoalteromone A exerts its anti-melanogenesis activity through effects on melanogenic genes. We further evaluated the skin-whitening effect of pseudoalteromone A in the three-dimensional (3D) pigmented-epidermis model, MelanoDerm, and visualized the 3D distribution of melanin by two-photon excited fluorescence imaging in this human skin equivalent. Collectively, our findings suggest that pseudoalteromone A inhibits tyrosinase activity and expression and that this accounts for its anti-melanogenic effects in melanocytes.

## 1. Introduction

There is little doubt that UV-radiation affects all people in their daily life. Harmful effects of solar radiation cause skin damage and skin aging, but the body counters these harmful influences through the production of the pigment, melanin [1,2]. Despite the considerable virtue of melanin, irregular production of this pigment underlies several skin diseases and conditions, including melasma, leukoplakia, lentigo, albinism, moles, and freckles [3]. To address the consequences of abnormal melanogenesis processes, numerous researchers have investigated melanogenesis mechanisms. The shared mechanism of melanin production is initiated as an oxidative progress in which tyrosine is converted first to 1-3,4-dihydroxyphenylalanine (DOPA), and then to dopaquinone, dopachrome and finally the pigment melanin, primarily by tyrosinase and related enzyme systems [4]. Given that catalysis is central to the oxidative action of tyrosinase, the capacity to suppress tyrosinase activity and expression is important for anti-melanogenesis activity. A number of medications that possess this capacity, such as kojic acid, 4-n-butylresorcinol, hydroquinone and tretinoin, are currently available [5]. Although these synthetic agents are very effective, they are accompanied by undesirable side-effects. For example, hydroquinone causes blistering, skin cracking, and dryness in individuals with sensitive skin. Therefore, recent research on anti-melanogenesis in the cosmetic industry has focused on the development of novel, natural sources-derived agents that are free from adverse effects. 

**Figure 1:**
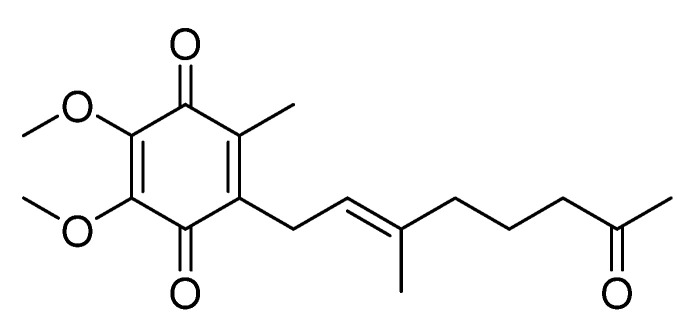
The structure of pseudoalteromone A (**1**).

Marine microorganisms are known to produce many bioactive molecules, with additional secondary metabolites exhibiting significant activities in various molecular and biochemical pathways [6]. In our continuing research effort to discover anti-melanogenic agents from marine microorganisms for use as constituents of cosmetic products, we have focused on screening crude extracts from libraries of cultures produced by dozens of marine microorganisms. In the process, we discovered that crude extracts of *Pseudoalteromonas* spp., APmarine002 and ROA-050, exhibit moderate inhibitory activity on melanogenesis in α-melanocyte-stimulating hormone (α-MSH) stimulated B16 melanoma cells. Using assay-guided purification of crude extracts of APmarine002 and ROA-050 based on anti-melanogenic activity, we identified the pseudoalteromone A (**1**) and *p*-coumaric acid as such anti-melanogenic agents (Figure 1). Pseudoalteromone A, first isolated from *Pseudoalteromonas* sp. CGH2XX and subsequently isolated from two other *Pseudoalteromonas* spp., *Pseudoalteromonas rubra* QD1-2 and *Pseudoalteromonas* sp. P1-9 [7,8], has been reported to exhibit cytotoxicity against MOLT-4 human acute lymphoblastic leukemia cells and inhibit the release of elastase by human neutrophils [9]. However, anti-melanogenic effects of pseudoalteromone A have not been previously reported. Here, we describe the inhibitory effects of pseudoalteromone A on melanogenesis both in vitro and in a 3D pigmented-epidermis model (MelanoDerm).

## 2. Results

### 2.1. Identification of Pseudoalteromone A

**Figure 2:**
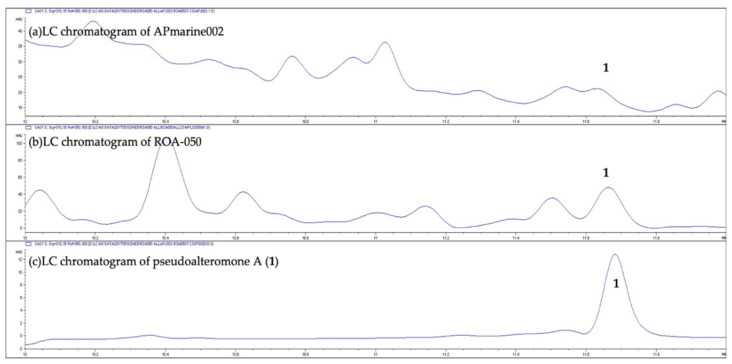
The LC analysis of the extract of APmarine002, the extract of ROA-050 and pseudoalteromone A.

**Figure 3:**
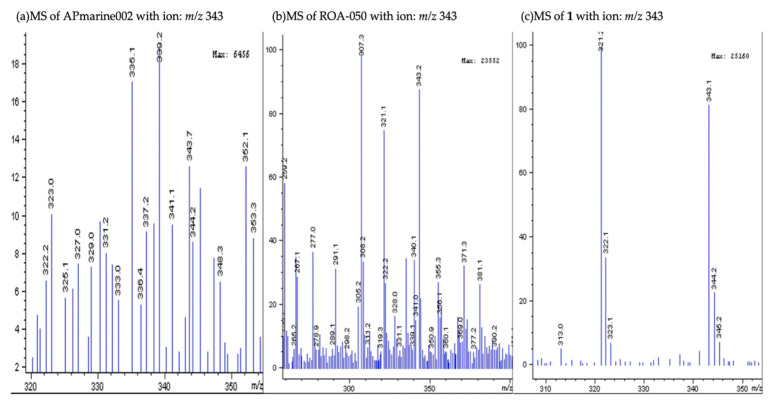
The MS spectrum of *m*/*z* 343 in the extract of APmarine002, extract of ROA-050 and pseudoalteromone A.

A liquid chromatography-mass spectrometry (LC-MS) analysis of crude extracts of culture broths of APmarine002 and ROA-050 revealed that both strains produce *p*-coumaric acid, which has been reported to exhibit anti-melanogenic effects through the inhibition of tyrosinase activity [10]. Interestingly, we found that the non-polar fraction, separate from the polar fraction containing *p*-coumaric acid-based anti-melanogenic activity, also exerted potent inhibitory activity on melanogenesis. Therefore, we intensively investigated the chemical components of the non-polar fraction to identify the distinctive natural products with the observed anti-melanogenic efficacy. The expanded LC-MS analysis of crude extracts of culture broths of APmarine002 and ROA-050 also revealed that both strains exhibited a peak with a retention time of 11.65 min and each peak showed the *m*/*z* 343 [M + Na]^+^ in the MS spectra (Figure 2 and Figure 3). 

Assay-guided isolation of the extract from large-scale cultures of ROA-050 yielded compound **1**. The ^1^H nuclear magnetic resonance (NMR) spectrum (CD_3_OD-*d*_4_, 400 MHz) of **1** showed two methoxy groups (δ_H_ 3.96 and 3.97), two vinyl methyl groups (δ_H_ 1.74 and 2.00), one acetyl methyl group (δ_H_ 2.09), an olefinic proton (δ_H_ 4.96), and four methylene groups (δ_H_ 1.64, 1.74, 2.40 and 3.21). The ^13^C NMR spectrum revealed 18 carbons, including three ketone carbonyls (δ_C_ 185.4, 186.0, and 211.9), five sp^2^ quaternary carbons (δ_C_ 138.0, 140.2, 142.7, 145.8 and 145.9), a sp^2^ methine (δ_C_ 121.3) and four sp^3^ methylenes (δ_C_ 12. 0, 1 6.1, 29.9, 61.5 and 61.6). 

A comparison of ^1^H, ^13^C NMR and MS spectra with previously reported data [9] identified compound **1** as pseudoalteromone A (Figures S1 and S2).

### 2.2. Anti-Melanogenic Efficacy of Pseudoalteromone A in B16, Melan-a and MNT-1 Cells

**Figure 4:**
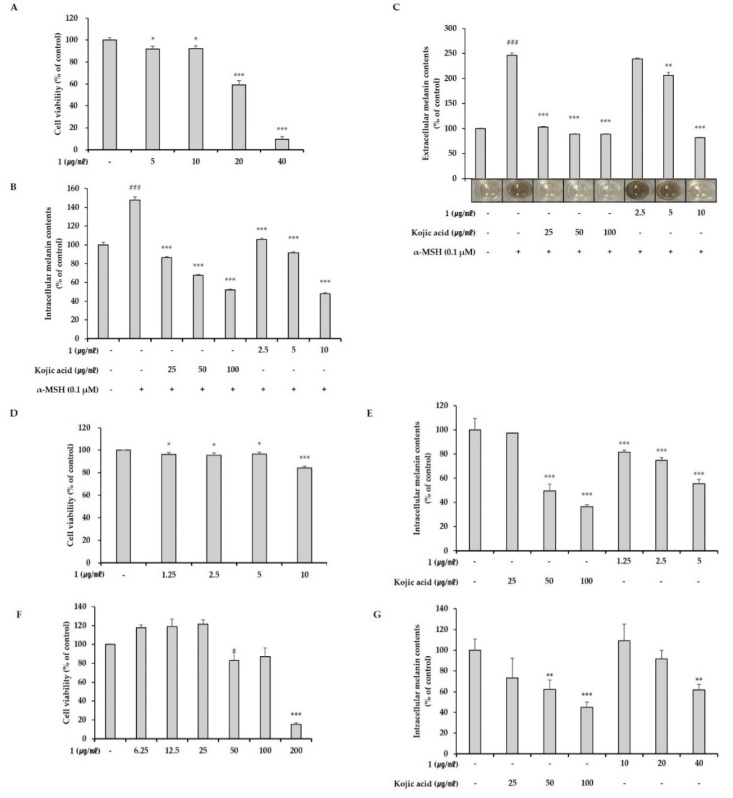
(**A**) Effect of pseudoalteromone A on B16 cell viability. B16 cells were treated with the indicated concentrations of pseudoalteromone A for 72 h, then the cell viability was estimated using CCK-8 assays. (**B**,**C**) Effects of pseudoalteromone A and kojic acid on intracellular and extracellular melanin content in α-MSH-stimulated B16 cells treated with the indicated concentrations of pseudoalteromone A and kojic acid for 72 h. (**B**) Intracellular melanin content was determined by measuring absorbance of B16 cell lysates at 405 nm and normalizing values to protein quantity, determined using a protein assay kit. (**C**) Extracellular melanin content determined colorimetrically. Photographs show colors of medium in each well following treatment with α-MSH and pseudoalteromone A. Results are presented as means ± SD, expressed as a percentage relative to the control group. (**D**,**F**) Viability of Melan-a cells (**D**) and MNT-1 human melanoma cell (**F**) after treatment with pseudoalteromone A for 72 h. (**E**,**G**) Representative results showing melanin content in Melan-a cells (**E**) and MNT-1 cells (**G**), measuring after treating with pseudoalteromone A or kojic acid for 72 h. Figures depict results representative of at least three replicate experiments. Results are presented as means ± SD, expressed as a percentage relative to the control group (* *p* < 0.05, ** *p* < 0.01, *** *p* < 0.001 vs. control group). “-“means non-treated or without any treatment and “+” means treated with the indicated compound (α-MSH (0.1 μM)). ### *p* < 0.001.

Before assessing the anti-melanogenic efficacy of pseudoalteromone A, we measured its cytotoxicity towards B16 cells using CCK-8 assays. B16 cells were cultured for 24 h and then treated with pseudoalteromone A at concentrations of 5–40 μg/mL for 72 h. As shown in Figure 4A, pseudoalteromone A was not cytotoxic at concentrations of 5–10 μg/mL. Accordingly, we used 2.5–10 μg/mL pseudoalteromone A in the subsequent experiments.

The maximum cytotoxicity-free concentration of pseudoalteromone A in B16 cells was 10 μg/mL. Thus, to assess the effect of pseudoalteromone A on the melanogenesis in B16 cells, we co-treated α-MSH-stimulated cells with pseudoalteromone A at concentrations of 2.5–10 μg/mL. After 72 h, we confirmed the increased production of α-MSH-stimulated cells compared with controls that were not treated with α-MSH and showed that both pseudoalteromone A and kojic acid, an anti-melanogenesis agent included as a positive control, inhibited, not only α-MSH-induced intracellular melanin synthesis (Figure 4B), but also extracellular melanin levels (Figure 4C) in B16 cells, effects that were concentration dependent. We further assessed the effects of pseudoalteromone A on the viability of a Melan-a mouse melanocyte cell and a MNT-1 human malignant melanoma cell as described for B16 cells, and found that pseudoalteromone A showed cytotoxicity toward Melan-a cells (Figure 4D) beginning at a concentration of 10 μg/mL and against MNT-1 at 50 μg/mL (Figure 4F). Therefore, for subsequent experiments, we selected 5 μg/mL and 40 μg/mL as sub-toxic concentrations of pseudoalteromone A for use in Melan-a cells and MNT-1 cells, respectively. Treatment of α-MSH-stimulated Melan-a cells and MNT-1 cells with pseudoalteromone A reduced intracellular melanin content in a concentration-dependent manner without causing cytotoxicity in either cell line. Specifically, treatment of Melan-a cells with pseudoalteromone A at a concentration of 5 μg/mL for 72 h reduced intracellular melanin content by 44.68% (Figure 4E), and at 40 μg/mL reduced melanin content in MNT-1 cells by 38.24% after 72 h (Figure 4G). Treatment with the positive control kojic acid at 25–100 μg/mL also decreased intracellular melanin content. However, these anti-melanogenic effects were lower than those of pseudoalteromone A under the same concentration conditions, indicating that pseudoalteromone A exerts a greater inhibitory effect than kojic acid in the present study.

**Figure 5:**
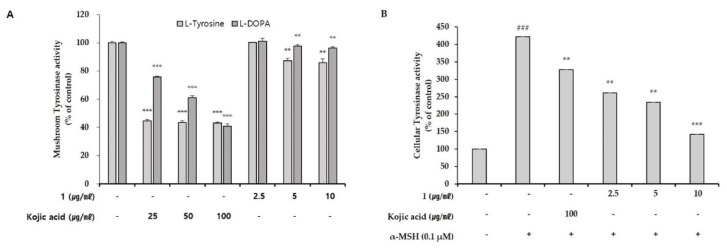
(**A**) Mushroom tyrosinase activity and (**B**) cellular tyrosinase activity were measured spectrophotometrically at 475 nm as indicated in the materials and methods. Results are presented as means ± SD, expressed as a percentage relative to the control group (** *p* < 0.01, *** *p* < 0.001 vs. control group or α-MSH-treated group; ### *p* < 0.001 vs. the α-MSH-untreated control group). “-“means non-treated or without any treatment and “+” means treated with the indicated compound (α-MSH (0.1 uM)).

The enzymatic activity tyrosinase is essential in the regulation of melanogenesis. Thus, inhibitory effect on this activity of the enzyme could explain the anti-melanogenic effect of pseudoalteromone A. To directly estimate the inhibitory effect of pseudoalteromone A on tyrosinase activity, we tested it against two different sources of tyrosinase: mushroom tyrosinase and cellular tyrosinase extracted from B16 cells. As shown in Figure 5A, pseudoalteromone A inhibited mushroom tyrosinase activity in a concentration-dependent manner. In this assay, we used L-tyrosine and L-3,4-dihydroxyphenylalanine (L-DOPA) as substrates to check both the tyrosine hydroxylase activity and DOPA oxidase activity. To more precisely examine the tyrosinase-inhibitory efficacy of pseudoalteromone A, we determined intracellular tyrosinase activity by measuring the oxidation rate of L-3,4-dihydroxyphenylalanine (L-DOPA) to dopachrome in B16 cells incubated with or without pseudoalteromone A. This analysis showed that pseudoalteromone A inhibited cellular tyrosinase activity in a concentration-dependent manner (Figure 5B).

**Figure 6:**
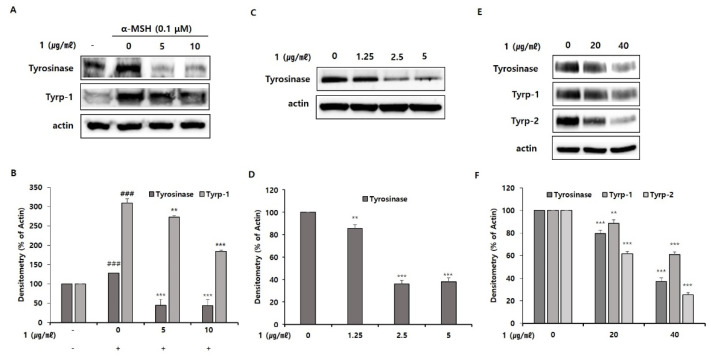
Inhibitory effect of pseudoalteromone A on the expression of melanogenic enzymes. (**A**,**C**,**E**) Protein levels of melanogenic enzymes were determined by Western blot analysis in B16 cells (1), Melan-a cells (**C**) and MNT-1 (**E**). Cells were treated with pseudoalteromone A for 72 h. Equal amounts of proteins from each cell type were resolved by SDS-PAGE on 10% gels and detected using specific antibodies; β-actin was detected as a control for equal loading. (**B**,**D**,**F**) Protein bands were quantified densitometrically and analysed using ImageJ software. Results for B16 cells (**B**), Melan-a cells (**D**) and MNT-1 cells (**F**) are presented as means ± SD, expressed as a percentage relative to controls (** *p* < 0.01, *** *p* < 0.001 vs. control group).; ### *p* < 0.001 vs. the α-MSH-untreated control group).

To investigate the effects of pseudoalteromone A on expression levels of melanogenesis-related protein, tyrosinase, Tyrp-1 and Tyrp-2, we performed Western blotting. This analysis showed that pseudoalteromone A decreased protein levels of tyrosinase and Tyrp-1 in B16 cells (Figure 6A,B); it also dramatically inhibited expression of tyrosinase in Melan-a cells (Figure 6C,D) and tyrosinase, Tyrp-1 and Tyrp-2 in MNT-1 cells (Figure 6E,F). Collectively, these results suggest that the anti-melanogenic effects of pseudoalteromone A are mediated by downregulation of protein levels of melanogenic genes. 

### 2.3. Whitening Effect of Pseudoalteromone A on a 3D Human Skin Equivalent

**Figure 7:**
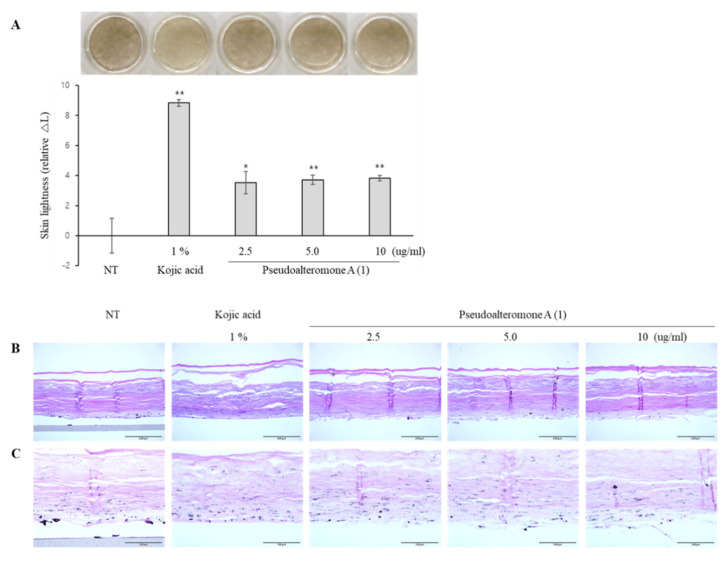
Skin whitening effects of pseudoalteromone A on a 3D pigmented epidermis skin model. MelanoDerm was treated with different concentrations of pseudoalteromone A for 14 days, and epidermal pigmentation was assessed by optical (**A**) and histological (**B**,**C**) analyses. Epidermal pigmentation was evaluated based on ΔL value, and results are presented as means ± SD, expressed as a percentage relative to controls (* *p*< 0.05, ** *p* < 0.01 vs. control group). The size of scale bar is 200 μm (**B**) and 100 μm (**C**).

**Figure 8:**
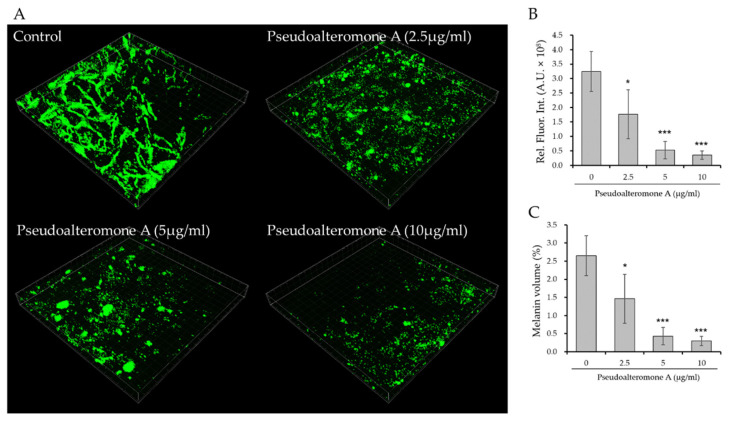
Anti-melanogenic effect of pseudoalteromone A on the human skin equivalent, MelanoDerm. (**A**) Label free visualization of melanin (green) in MelanoDerm tissue by TPEF microscopy. The measured volume was 400 (x) × 400 (y) × 40 (z) μm^3^. (**B**,**C**) Quantification of relative TPEF intensities (**B**) and melanin volume (**C**) in five different regions. Results are expressed as means ± SD (* *p* < 0.05, *** *p* < 0.001 vs. control group).

To further investigate the anti-melanogenic efficacy of pseudoalteromone A, we evaluated its skin whitening effect on MelanoDerm (MEL-300-B, MatTek Corp., Ashland, MA, USA), a 3D pigmented epidermis model. MelanoDerm was cultured in the presence or absence of pseudoalteromone A for 14 days, and the skin-whitening effect was evaluated through optical and histological examinations. The activity of melanocytes causes MelanoDerm to spontaneously darken according to the duration of the incubation period. Notably, pseudoalteromone A-treated cultures were brighter than non-treated controls, a finding confirmed by the demonstration of a significant lightening effect, measured by image analysis as a change in skin lightness (ΔL) (Figure 7A). We were also able to confirm the whitening effect of pseudoalteromone A through histological examination (Figure 7B,C). Fontana Masson’s (F-M) staining revealed that pseudoalteromone A treatment significantly reduced epidermal melanin, and hematoxylin and eosin (H&E) staining showed no significant epidermal damage. These results demonstrate the potential skin whitening effect of pseudoalteromone A through inhibition of epidermal pigmentation. Furthermore, visualization of the 3D distribution of melanin in the basal layer of MelanoDerm by TPEF microscopy (Figure 8A) confirmed that the extent of melanin decrease depended on the pseudoalteromone A concentration (Figure 8B,C).

## 3. Discussion

We showed that two different marine-derived *Pseudoalteromonas* strains, APmarine002 (isolated from soft coral in Jeju island) and ROA-050 (isolated from marine sediments of Pohang, South Korea), produced the same anti-melanogenic agents, pseudoalteromone A (**1**) and *p*-coumaric acid. We also demonstrated that pseudoalteromone A clearly inhibits melanin synthesis in different cell lines (B16, Melan-a and MNT-1 cells) and exerts a whitening effect in a human skin equivalent. 

To begin defining the action mechanism of pseudoalteromone A, we tested the inhibitory effect of pseudoalteromone A on tyrosinase activity. First, we determined whether pseudoalteromone A directly inhibits tyrosinase activity. Simultaneously, we performed the total cellular tyrosinase activity after pseudoalteromone A treatment in α-MSH-treated B16 cells. In Figure 5A, we found that the de-pigmentary effect of pseudoalteromone A on melanocytes mainly depends on reduced tyrosinase activity, as supported by the significant (albeit weak) direct inhibition of mushroom tyrosinase activity and robust inhibition of cellular tyrosinase activity by pseudoalteromone A (Figure 5). Based on above data, we supposed that the decreased total cellular tyrosinase activity by pseudoalteromone A treatment may be mainly caused by the decreased level of tyrosinase expression rather than the direct inhibition of tyrosinase activity. In addition, further support is provided by the observation that pseudoalteromone A reduced expression of tyrosinase as well as that of the tyrosine-related enzymes, Tyrp-1 and Tyrp-2 (Figure 6). Collectively, these findings suggest that pseudoalteromone A inhibits melanogenesis by suppressing both tyrosinase activity and expression. 

Despite finding that pseudoalteromone A suppressed tyrosinase, Tyrp-1 and Tyrp-2 expression, our evidence for an underlying molecular action mechanism of pseudoalteromone A is incomplete. To establish a detailed molecular mechanism, we plan future studies to determine whether pseudoalteromone A suppresses expression of melanocyte-inducing transcription factor (MiTF) through inhibition of mitogen-activated protein kinase (MAPK) or cAMP-depend on protein kinase (PKA) signaling pathways in melanocytes, reflecting the fact that MAPK and PKA signaling are intimately linked with MiTF-induced tyrosinase expression during melanogenesis [11,12,13,14,15,16,17]. Collectively, the data from this study provide preliminary evidence supporting the possibility of pseudoalteromone A as a potent and effective whitening agent that could be used in cosmetics and medicinal formulations. 

## 4. Materials and Methods

### 4.1. General Experimental Procedures

UV spectra were recorded in methanol (MeOH) on a Cinco UVS-2100 spectrophotometer. 1D and 2D NMR spectra were recorded on a Varian Unity-Enova at 400 MHz using signals of the residual solvent as internal references (p_h._ 3.31 ppm and a_ct_ 49.00 ppm for methanol-*d*_4_ (CD_3_OD-*d*_4_). Low-resolution LC/MS measurement was performed at the National Research Facilities and Equipment Center (Nano Bioenergy Materials Center) at Elwha Woman’s University using an Agilent Technologies 1260 quadrupole and Waters micromassZQ LC/MS system with a reversed-phase column (Phenomenex Luna C18 (2) 100 Å, 50 mm × 4.6 mm, 5 µm) at a flow rate 1.0 mL/min. Column chromatography separation was performed using a C18 column eluting with a gradient of MeOH and H_2_O. Fractions were purified using a WATERS 1525 binary HPLC pump with a reversed-phase C18 column (Phenomenex Luna C18 (2), 250 mm × 10 mm, 5 μm) eluting with 48% CH_3_CN in H_2_O at flow rate of 2.0 mL/min.

### 4.2. Strain Isolation

*Pseudoalteromonas* strain APmarine002 was isolated from coral collected at Jeju island. *Pseudoalteromonas* strain ROA-050 was isolated from the marine sediments of Pohang, South Korea. An NCBI blast analysis of 16S rRNA gene sequences identified Strain APmarine002 as *Pseudoalteromonas rubra* QD1-2 and strain ROA-050 as *Pseudoalteromonas piscicida,* with 100.0% identity in both cases. 

### 4.3. Fermentation, Extraction and Purification

Strains APmarine002 and ROA-050 were independently cultured in 1 × 2.5 L Ultra Yield Flasks, each containing 1 L of seawater-based medium (10 g starch, 2 g yeast extract, 4 g peptone, 34.75 g artificial sea salt dissolved in distilled H_2_O), and at 27 °C with shaking (150 rpm). After 7 days of cultivation, each broth was extracted using ethyl acetate (EtOAc; 1 L overall), and the solvent was removed in vacuo to yield 23.1 mg and 84.2 mg of EtOAc crude extract, respectively. Both extracts were used to perform the LC-MS analysis. For the large cultivation to isolate compound **1**, strain ROA-050 was cultured in 40 × 2.5 L Ultra Yield Flasks, each containing 1 L of seawater-based medium (10 g starch, 2 g yeast extract, 4 g peptone, 34.75 g artificial sea salt dissolved in distilled H_2_O), and at 27 °C with shaking (150 rpm). After 7 days of cultivation, the broth was extracted using ethyl acetate (EtOAc; 40 L overall), and the solvent was removed in vacuo to yield 3.5 g of an EtOAc crude extract. The extract was fractionated by open column chromatography on a C-18 resin using a step gradient elution of H_2_O and MeOH, yielding eight fractions. Fraction 4 and 5, eluted with 60~80% of MeOH in H_2_O (770.4 mg), was further purified by reversed-phased HPLC under isocratic conditions with 48% aqueous CH_3_CN to yield **1** (17.3 mg).

*Pseudoalteromone A* (**1**): orange oil, UV (MeOH) *λ_max_* (log ε) 200 (2.15), 281 (2.09) and 262 (1.24); LRESIMS *m*/*z* 343.1 [M + Na]^+^.

### 4.4. Cell Culture

B16 cells, mouse melanoma cell line, were cultured in Dulbecco’s modified Eagle’s medium (DMEM; Welgen, Korea) containing 5% fatal bovine serum (FBS; ATCC, Manassas, VA, USA) and 1% penicillin-streptomycin mixture (Lonza, Basel, Switzerland). Melan-a cells, an immortalized mouse melanocyte cell line, were grown in RPMI-1640 medium (Lonza, Walkersvile, MD, USA) supplemented with 10% FBS (Gibco, Grand Island, NY, USA), 1% penicillin-streptomycin mixture and 200 nM phorbol 12-myristate 13-acetate (PMA; Sigma-Aldrich, St. Louis, MO, USA). MNT-1 cells, a human melanoma cell line, were cultured in Minimum Essential Medium (MEM; Gibco, Grand Island, NY, USA) containing 1% DMEM (Welgen, Korea), 20% FBS, 1% penicillin-streptomycin mixture and 1% N-(2-hydroxyethyl)piperazine-N’-(2-ethanesulfonic acid) (HEPES 1 M; Gibco, Taiwan). All cells were cultured at 37 °C in a humidified environment with 5% CO_2_/95% air environment.

### 4.5. Cell Viability

B16 cells, Melan-a cells and MNT-1 cells were seeded on 96-well plates (1 × 10^4^ cells/well) and cultured for 24 h, after which the medium was replaced with the medium containing pseudoalteromone A, diluted to the indicated concentrations, and plates were incubated for 72 h. The medium was then removed and the fresh culture medium containing 10% CCK-8 solution (DOJINDO, Tokyo, Japan) was added. After incubating 1 h, supernatants of each cell were transferred to 96-well plates, and the absorbance was measured at 450 nm using a Synergy™ HTX Multi-Mode Microplate Reader (Bioteck, Winooski, VT, USA).

### 4.6. Measurement of Melanin Content

B16 cells were seeded overnight in 48-well plates (2 × 10^4^ cells/well) and then treated for 72 h with increasing concentrations of pseudoalteromone A. The extracellular melanin content in the phenol red-free cell culture medium was measured at 405 nm with Synergy™ HTX Multi-Mode Microplate Reader (Bioteck, Winooski, VT, USA). After removing the medium, 200 μL of 1 N NaOH was added, and plates were heated to 60 °C for 30 min to solubilize intracellular melanin. Next, 100 μL aliquots of lysate were transferred to a 96-well plate and absorbance was measured at 405 nm. Melan-a cells and MNT-1 cells were seeded in 24-well plates (10 × 10^4^ cells/well) for 24 h, and treated with pseudoalteromone A. Then, the intracellular melanin contents were measured. Results were normalized to total protein content, determined using a Pierce BCA Protein Assay Kit (Thermo Fisher Scientific, Waltham, MA, USA). Intracellular melanin levels are presented relative to those in the control group, expressed as a percentage.

### 4.7. Cell-Free Enzymatic Assay for Tyrosinase Activity

The inhibitory effects of pseudoalteromone A on tyrosinase activity, tested using a mushroom tyrosinase enzyme, was assessed as a function of DOPA oxidase activity and t and tyrosine hydroxylase activity. For these experiments, a 5 μL sample was added to a 96-well plate to which was added 50 μL of 0.1 M sodium phosphate buffer (pH 6.8), 40 μL distilled water, and 5 μL of enzyme solution (2000-unit mushroom tyrosinase). After mixing, 50 μL of 20 mM L-DOPA or 0.03% L-tyrosine (tyrosinase substrate) was added to each well and the amount of products formed in the reaction mixture was determined by kinetically measuring absorbance values at 475 nm at 10 min intervals for at least 1 h at 37 °C using a spectrophotometer.

### 4.8. Assay of Cellular Tyrosinase Activity

The activity of tyrosinase enzyme was evaluated by measuring the oxidase rate of L-DOPA to dopachrome, an intermediate in melanin biosynthesis. B16 melanoma cells were plated in 6-well plates (20 × 10^4^ cells/well) and incubated overnight, then treated with increasing concentrations of pseudoalteromone A for 3 days in a humidified incubator. Cells were then washed twice in ice-cold phosphate-buffered saline (PBS) and lysed in 1 mL of 0.1 M sodium phosphate buffer (pH 6.8) containing 1% (*w*/*v*) Triton X-100. The resulting whole-cell lysates were microcentrifuged at 13,000 rpm for 20 min at 4 °C, and the resulting supernatants were placed in a 96-well plate for reactions with L-DOPA (2 mg/10 mL). Tyrosinase activity was analyzed spectrophotometrically by kinetically measuring absorbance values at 475 nm at 10 min intervals for at least 1 h at 37 °C using a spectrophotometer.

### 4.9. Western Blotting

B16, Melan-A and MNT-1 cells were seeded onto 6-well plates (20 × 10⁴ cells/well) and incubated for 24 h. The medium was then removed and fresh culture medium containing different concentrations of pseudoalteromone A was added, after which plates were incubated for the appropriate time. After washing twice with ice-cold PBS, intracellular proteins were extracted from each cell type by lysing cells in 1 × RIPA buffer (Cell Signaling Technology, Danvers, MA, USA), diluted with distilled water containing protease inhibitor cocktail III and phosphatase inhibitor Cocktail set III (Merck, Darmstadt, Germany) at a ratio of 200:1. Cell lysates were centrifuged at 13,000 rpm at 4 °C, and protein concentration was measured using a Pierce BCA Protein Assay Kit (Thermo Fisher Scientific, Waltham, MA, USA). Appropriate amounts of protein from each cell type were resolved by SDS-PAGE on 10% gels and then transferred onto a nitrocellulose membrane (Bio-Rd, Hercules, CA, USA). Membranes were blocked by incubating in 1 × Tris-buffered saline (TBS) containing 5% skim milk for at least 1 h, then incubated at 4 °C overnight with primary antibodies, diluted as recommended by the manufacturer. Membranes are washed three times in TBS containing Tween 20 and incubated with species-appropriate secondary antibodies at recommended dilutions for 1 h at room temperature. Immuno-reactive proteins were visualized using Clarity™ Western ECL substrate (Bio-Rad, Hercules, CA, USA). The densities of target protein bands were measured using iBright™ CL750 Imaging System (Invitrogen, Carlsbad, CA, USA).

### 4.10. Skin-Whitening Assay Using a Pigmented 3D Skin Model

MelanoDerm (MEL-300-B, MatTek Corp., Ashland, MA, USA), used for the skin-lightening study, was cultured in EPI-100-NMM-113-PRF medium (MatTek Corp., Ashland, MA, USA) at 37 °C in a humidified 5% CO_2_ incubator. Different concentrations of pseudoalteromone A were added to the culture medium every other day for 14 days. Thereafter, epidermal pigmentation was examined by performing optical and histological analyses. The epidermal pigmentation level was calculated by comparing variations in L* values (a lightness/darkness index) on days 1 and 14 and estimating the difference between them (ΔL). For the histological examination, tissues were fixed in 10% neutral buffered formalin (BBC Biochemical, Mount Vernon, WA, USA) and embedded in paraffin. Paraffin-embedded samples were then sliced into 5 μm sections, stained with hematoxylin and eosin (H and E) and Fontana Masson’s (F-M) staining (for melanin), and then examined histologically.

### 4.11. Two-Photon Excitation Fluorescence (TPEF) Imaging

TPEF imaging was used to visualize the three-dimensional distribution of melanin in the human skin equivalent, as described in our previous studies [18,19]. Intracellular melanin (green in Figure 8) in the melanocyte-rich layer was measured by imaging the basement membrane side of MelanoDerm samples in a total volume of 400 (x) × 400 (y) × 40 (z) μm^3^. Relative TPEF signal intensities and melanin volume were quantified using Image-Pro Premier 3D software (Media Cybernetics, Inc., Bethesda, MD, USA).

## 5. Conclusions

In this paper, we identified a new anti-melanogenic agent, pseudoalteromone A, from cultures of the marine microorganism, *Pseudoalteromonas* spp. APmarine002 and ROA-050. We showed that pseudoalteromone A suppresses melanin synthesis in vitro, reducing tyrosinase activity and expression, and confirmed its skin-whitening effect in MelanoDerm, a 3D pigmented epidermis model. On the basis of these results, we conclude that marine microorganisms are a good source of biologically active compounds and further suggest that pseudoalteromone A is a promising anti-melanogenic agent in the cosmetic field.

## Data Availability

Not applicable.

## Supplementary Materials

The following are available online at https://www.mdpi.com/article/10.3390/md19110612/s1, Figure S1: ^1^H NMR spectrum of pseudoalteromone A (**1**), Figure S2: ^13^C NMR spectrum of pseudoalteromone A (**1**).
