# Supplementary material for: Pseudoalteromone A, a Ubiquinone Derivative from Marine Pseudoalteromonas spp., Suppresses Melanogenesis
Source: Mar Drugs. 2021 Oct 28;19(11):612. doi: 10.3390/md19110612 (PMC8618130; doi:10.3390/md19110612)
Supplement: Supplementary file 1 [file marinedrugs-19-00612-s001.zip › marinedrugs-1406387-supplementary.pdf]

Supporting Information for

Pseudoalteromone A, a Ubiquinone Derivative from Marine *Pseudoalteromonas* spp., Suppresses Melanogenesis.

Su Jin Lim [1,†], Daejin Min [2,†], Sohee Kim [3,†], Jihye Lee [4,5], Eun-Soo Lee [2], Hyuk Kim [2], Sung Yoen Cho [2], Heung Soo Beak [2], Chang Seok Lee [1,*], Sang-Jip Nam [4,*] and Jaeyoung Ko [2,*]

[1] Department of Beauty and Cosmetic Science, Eulji University, Seongnam 13135, Republic of Korea; jimnote91@naver.com (S.J.L)

[2] Basic Research & Innovation Division Amorepacific R&D Unit, Yongin 17074, Republic of Korea; djmin@amorepacific.com (D.M.), soopian82@gmail.com (E.-S.L.), atharabia@amorepacific.com (H.K.), csy123@amorepacific.com (S.Y.C.), monmimi@amorepacific.com (H.S.B.)

[3] The Graduate School of Industrial Pharmaceutical Sciences, Ewha Womans University, Seoul 03760, Republic of Korea; chi0736@naver.com (S.K)

[4] Department of Chemistry and Nanoscience, Ewha Womans University, Seoul 03760, Republic of Korea, Korea; jl3414@gmail.com (J.L)

[5] Laboratories of Marine New Drugs, REDONE Seoul, Seoul 08594, Republic of Korea

* Correspondence: cslee2010@eulji.ac.kr (C.S.L), sjnam@ewha.ac.kr (S.-J.N.), jaeyoungko@amorepacific.com (J.K.)

† These authors contributed equally to this work

Table of Contents

Figure S1 ^{1}H NMR spectrum (300 MHz, CD_3OD) of pseudoalteromone A (**1**) ········ **S3**

Figure S2 ^{13}C NMR spectrum (125 MHz, CD_3OD) of pseudoalteromone A (**1**) ········ **S4**

Table S1 Experimental (A) and reference (B) ^{1}H and ^{13}C NMR data of pseudoalteromone A (**1**) ········ **S5**

Figure S1 ^{1}H NMR spec trum (400 MHz, CD_3O D) of pseudoalteromone A (**1**)

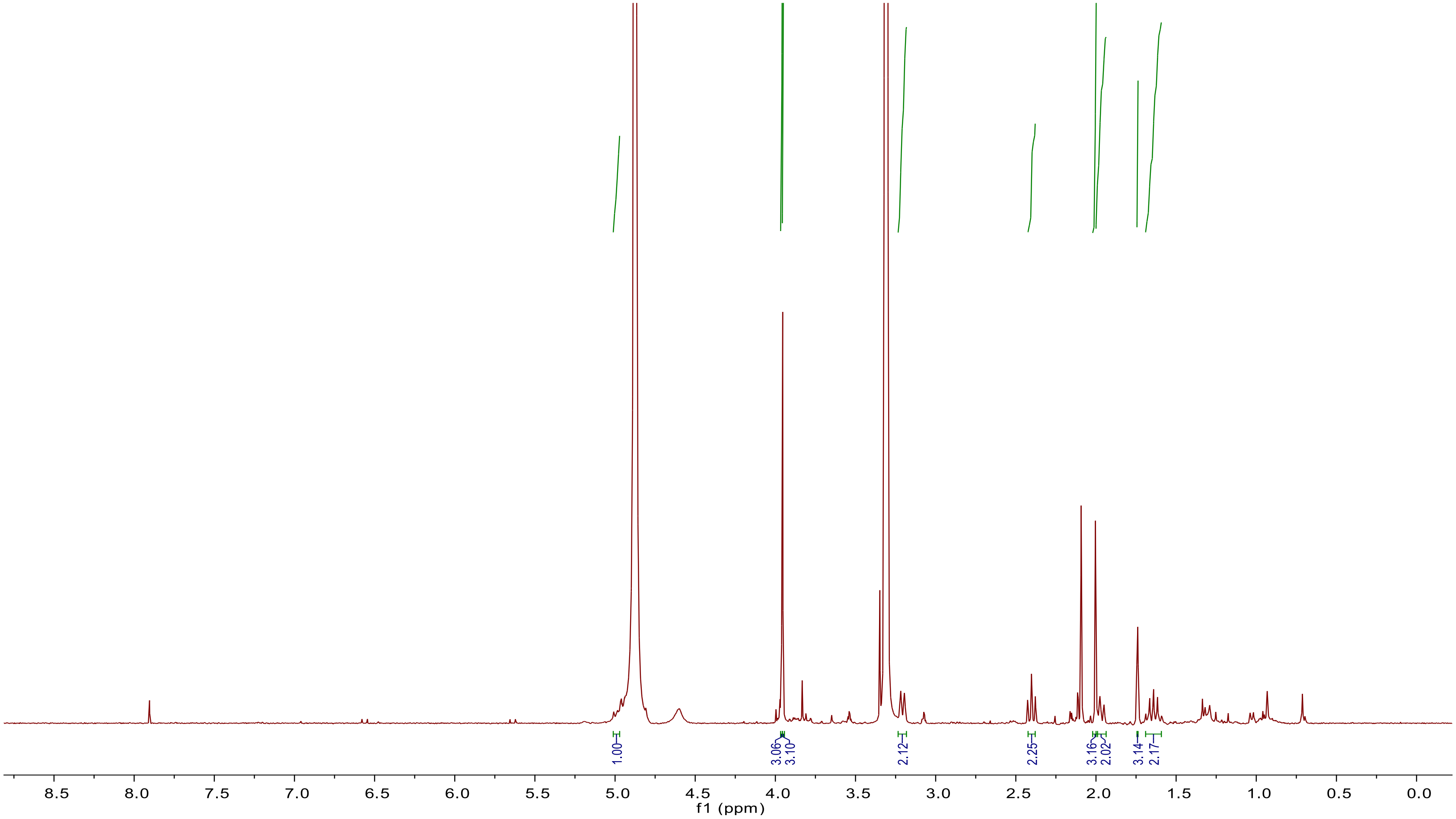

Figure S2 ^{13}C NMR spectrum (125 MHz, CD_3OD) of pseudoalteromone A (**1**)

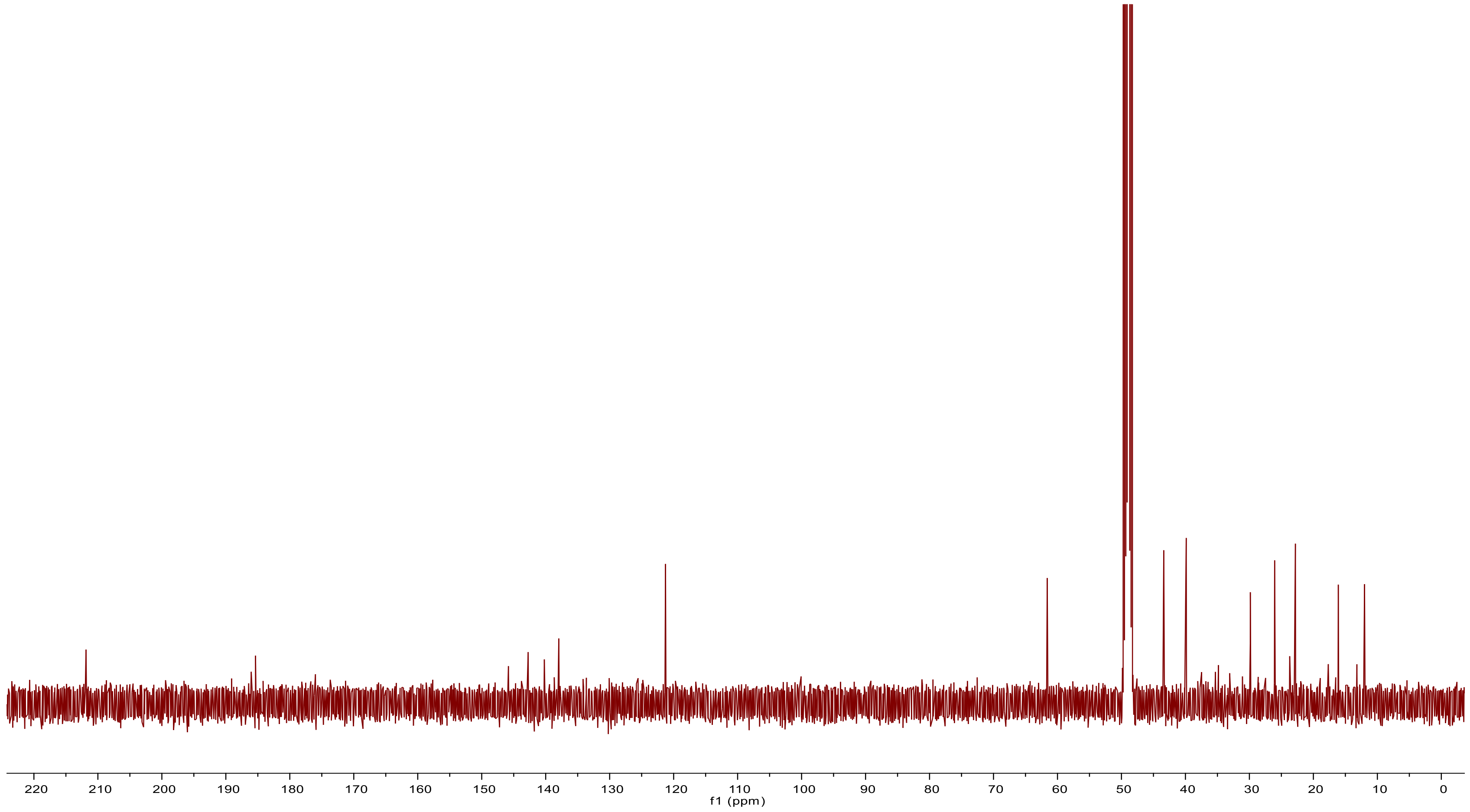

Table S1 Experimental (A) and reference (B) ^{1}H and ^{13}C NMR data of pseudoalteromone A (**1**)

	A		B[a]	
No.	δ_C, mult.[b]	δ_H, (*J* in Hz)q [b, c]	δ_C, mult.[d]	δ_H, (*J* in Hz)[d]
1	26.1, CH_2	3.21, d (6.8)	26.0, CH_2	3.19, d (6.8)
2	121.3, CH	4.96, tq (6.8, 0.8)	121.3, CH	4.96, m
3	138.0, qC		138.0, qC	
4	39.9, CH_2	1.97, t (7.6)	39.9, CH_2	1.97, t (7.5)
5	22.8, CH_2	1.64, q (7.6)	22.8, CH_2	1.64, q (7.6)
6	43.4, CH_2	2.40, t (7.6)	43.5, CH_2	2.39, t (7.6)
7	211.9, qC		211.8, qC	
8	29.9, CH_3	2.09, s	29.9, CH_3	2.09, s
9	16.1, CH_3	1.74, d (0.8)	16.1, CH_3	1.72, d (0.8)
1′	186.0, qC		186.2, qC	

2′	140.2, qC		140.3, qC	
3′	142.7, qC		142.7, qC	
4′	185.4, qC		185.4, qC	
5′	145.8, qC		145.9, qC	
6′	145.9, qC		146.1, qC	
7′	12.0, CH_3	2.00, s	12.0, CH_3	1.99, s
5′-OMe	61.5, CH_3	3.96, s	61.6, CH_3	3.96, s
6′-OMe	61.6, CH_3	3.97, s	61.7, CH_3	3.97, s

[a] Reference 7: Ding, L.; He, S.; Yan, X. Efficient preparation of pseudoalteromone A f from marine *Pseudoalteromonas rubra* QD1-2 by combination of response surface methodology and high-speed counter-current chromatography: a comparison with high-performance liquid chromatography. Mircobiol. Biotech. 2014, 98, 4369–4377.

[b] 400MHz for ^1H NMR and 125MHz for ^{13}C NMR in CD_3OD.

[c] Numbers of attached protons were determined by analysis of 2D spectra.

[d] 500MHz for ^1H NMR and 125MHz for ^{13}C NMR in CD_3OD.
